# Prior knowledge as a moderator between signaling and learning performance in immersive virtual reality laboratories

**DOI:** 10.3389/fpsyg.2023.1118174

**Published:** 2023-02-21

**Authors:** Jining Han, Geping Liu, Qiyu Zheng

**Affiliations:** ^1^Faculty of Education, Southwest University, Chongqing, China; ^2^Chaoyang School, Chongqing, China

**Keywords:** immersive virtual reality, signaling, prior knowledge, cognitive load, learning performance

## Abstract

The purpose of this study is to investigate the effects of signaling and prior knowledge on the cognitive loads, motivations, and learning of college students in an immersive virtual reality (IVR) environment. This study applied a 2 (signaling vs. no signaling) by 2 (high vs. low prior knowledge levels) between-subjects factorial design. The results revealed that signaling directed the attention of students with low prior knowledge levels, effectively helped them select relevant information and reduced their cognitive loads, whereas signaling had no significant effect on the cognitive loads, intrinsic motivation, and learning performance of learners with high levels of prior knowledge. These results suggest that IVR environments for students with low prior knowledge levels should reduce cognitive load and improve learning, and signals in the form of text annotations and color changes are recommended for additional support. Students with high prior knowledge levels do not require additional signals to support learning; therefore, the IVR environment needs to be designed in such a way as to be tailored to the individual differences of students.

## 1. Introduction

According to the European Commission’s Education and Training 2020 Strategy and the report on the agenda for the modernization of higher education, one of the main challenges for higher education is to improve the quality of teaching and learning. Studies show that there is a high demand for laboratory teaching in higher education, and due to limitations on staff and equipment that were induced by the COVID-19 pandemic, students have only limited access to equipment and machines in the laboratory courses offered in higher education (Mishra et al., 2020). By using virtual reality (VR) in class, students are able to directly observe experimental phenomena and independently master the learning process in a VR environment, increasing the effectiveness and efficacy of acquiring knowledge and skills (Jensen and Konradsen, 2018; Radianti et al., 2020). VR provides contextual and diverse learning resources, but it also inevitably brings new technological problems, such as the unreasonable presentation of visual elements and maladaptation with multichannel perception, which causes learners to experience increased cognitive loads and experience a negative impact (Baceviciute et al., 2021; Parong and Mayer, 2021b); therefore, instructional aid is needed to help learners.

With the increasing fidelity and visual richness of the teaching and learning environment, especially in immersive virtual reality (IVR) environments, the visual load for learners increases significantly (e.g., Birbara and Pather, 2021; Parong and Mayer, 2021a). Many studies have found that signaling can improve learning and assist learners in their visual search for important information (e.g., Johnson et al., 2014; Arslan-Ari, 2018; Albus et al., 2021), thereby reducing learning time. The learner’s prior knowledge is an important determinant of the learning effect (Johnson and Lawson, 1998). It was formerly widely assumed that learners constructed concepts based on their prior knowledge (Novak, 1990), but further research has revealed that prior knowledge affects not only learners’ subsequent conceptual learning but also their perception and attention (Kim and Rehder, 2011; Buckingham et al., 2016). Therefore, changes in the way learners interpret visual representations also largely depend on their prior knowledge. However, only a few studies have investigated the effects of learners’ different prior knowledge levels on the efficacy of signaling (e.g., Richter et al., 2016). At the same time, there is a lack of studies on how interactive IVR laboratories impact the cognitive processes of learners interacting with learning situations, which is an important perspective for research on VR technology in education.

## 2. Literature review

### 2.1. Virtual reality in multimedia learning

Mayer (2020) published the third edition of Multimedia Learning, and the principles of multimedia instructional design were reorganized into three sections. In the context of multimedia learning, the first section offers the principles for reducing extraneous processing, the second for managing essential processing, and the third for fostering generative processing (Mayer, 2020). With the rapid development of VR in education, many studies have discussed multimedia learning principles in VR environments and provided implications for designing VR for use in multimedia learning.

Makransky et al. (2019a) used the redundancy principle to design virtual learning resources in a study in which two groups of learners learned with either virtual reality head-mounted display (VR-HMD) or desktop presentation and received two learning simulations, one with on-screen text and one with on-screen text with narration. The study found that the redundancy principle had no effect on the learning process. In another study by Parong and Mayer (2018), an empirical study was conducted on the effectiveness of the segmenting principle. They compared the learning effect of a self-paced slideshow with that of a continuous VR animation. The learners who received the slideshow scored higher on the factual questions than the VR group learners but did not score as high on the conceptual questions. Baceviciute et al. (2021) explored the application of the modality principle in VR by comparing audio-visual and visual-only presentations, and the results showed an inverse modality effect, whereby learning under visual-only presentations was more effective than auditory and visual presentations. Petersen et al. (2020) investigated pretraining principles in VR. The experimental group received narrative pretraining followed by a VR exploration tour, while the control group went straight to VR learning. The pretraining group showed a significant increase in test scores for knowledge transfer. Makransky et al. (2019b) examined the embodiment principle and found that gender-specific design in VR learning environments can impact learning performance, retention, and transfer. Makransky et al. (2020) explored the formulating generative learning strategies in VR, and they found that compared to video, VR can better facilitate the transfer of procedural knowledge with generative learning strategies.

The transferability and effectiveness of multimedia design principles in IVR are visible in studies that show a relatively significant effect on learners in IVR learning environments, mainly reflected in cognitive processing and cognitive loads, but no clear conclusions can be drawn about the specific learning process at present (Han et al., 2021). More empirical findings are needed to further enrich the effectiveness of multimedia design principles on the design of IVR learning environments.

### 2.2. Signaling

Some studies have pointed out that adding visual cues to learning materials can reduce cognitive load and effectively improve learning effects (Mayer et al., 2003). The signaling principle, which promotes goal-oriented learning by highlighting the organizational structure, can be followed in the design and development of learning materials to enhance learning by highlighting important information (e.g., underlining, marking, etc.,) to attract learners’ attention (de Koning et al., 2009).

The salient visual cues in the traditional multimedia learning environment are the signaling models, which can be divided into two categories: textual signaling and graphic signaling (Van Gog, 2014). The experimental results of the studies that have been conducted show that the two forms of signaling have different effects on learners. For example, a study showed that when color flashes were used as signals to draw learners’ attention to various laboratory tools in the virtual environment, learners experienced a negative effect on their learning, and some color flashes had greater negative impacts than others (Jeung et al., 1997). Contrary to the findings of a previous study, another study found that the application of color coding in mind maps had a more positive effect on learning (Ferrara and Butcher, 2011). Hwang and Shin (2018) combined dynamic and static visualizations into one medium by adding transparent static images (graphical signals) to virtual animations. The results showed that combining dynamic and static visualization signals was beneficial in reducing the cognitive load of learners.

Due to the highly immersive feature of the IVR learning environment, the textual and graphic signals used are quite different from those designed and produced in traditional multimedia materials (Albus et al., 2021), resulting in a large difference in the users’ experience, and it is not yet possible to conclude with certainty whether the effect of textual or graphic signals in IVR environments is consistent with the findings of previous studies. Therefore, further research on the application of signaling in IVR experiments is needed to provide more diverse perspectives on the design of IVR environments to enhance the practicability and effectiveness of IVR technology.

### 2.3. Prior knowledge impacts the effect of signaling

Learners use their prior knowledge to select relevant information from visual cues and to find, retrieve and add information from their prior knowledge and finally construct mental models (Braune and Foshay, 1983). Because the signaling effect and difference in the level of prior knowledge are very significant and there is an interaction effect (Johnson et al., 2014; Arslan-Ari, 2018), it is necessary to explore the synergetic effect of signaling and prior knowledge level in the IVR environment.

Cook (2006) investigated the instructional design of visual representations in the science classroom, conducting empirical research, and integrating theoretical concepts related to cognitive load. The study concluded that individual differences—especially learners’ prior knowledge level—are a key factor in determining the impact of visual representations on learners’ cognitive structures and learning processes because prior knowledge can determine the ease with which learners perceive and interpret visual representations in working memory. Johnson et al. (2013, 2014) found that learners with low prior knowledge levels performed better with signals than without them, but signals were not very helpful for learners with high prior knowledge. They found that there was a significant interaction effect when the signals were presented together with an Animation Pedagogical Agent (APA) in the learning material; however, the arrow signals had no significant effect on learners when the learning material did not include an APA. Khacharem (2017) reported that red circle signals in static images of football matches were effective in directing the attention of novice learners; however, this strategy was ineffective for learners with relevant sports experience.

Arslan-Ari (2018) investigated the effects of signaling and prior knowledge levels on student learning from animations with narration, and the results showed that prior knowledge and signaling had significant interaction effects on the learning effect. The effect of providing signaling in instructional animations varies with learners’ prior knowledge levels. Specifically, when visual signals were provided, learners with low prior knowledge benefited more, while visual signals did not have an effect on learners with high prior knowledge. Vogt et al. (2020) investigated the effects of signals and graphic signals on learning performance and their intrinsic interaction mechanisms. The findings suggest that prior knowledge is an important moderating factor and that although learners with a low prior knowledge level can benefit from both types of signals, the gains in the learning effect they obtain are not significant, whereas learners with a moderate prior knowledge level benefited from the combined effect of both types of signals. For those with a higher prior knowledge level, signals can instead reduce their learning effect. Therefore, to deeply understand the role of the signaling principle in supporting or hindering different learners, it is necessary to conduct a more refined analysis of signal reception and cognitive construction during the learning process and to explore how learners obtain positive effects and cognitive feedback.

Although most studies have shown the moderating effect of prior knowledge level in multimedia learning, existing studies have not been concerned with the interaction between prior knowledge level and signaling design in IVR. The effects of visual selection, organization, and integration processes on the learning process in IVR environments also vary with individual learners; therefore, this study explores the interaction effect of signaling and prior knowledge level on learning effect by measuring learners’ cognitive load, intrinsic motivation, and learning performance in IVR. This study contributes to the empirical research on signaling principles in IVR laboratories in theory and practice and provides valuable information for instructional designers and practitioners.

### 2.4. Purpose of the study

The presentation of learning materials in highly IVR environments requires explicit signal selection and organization to assist learners in information reception and selection, including from text and images, in different sensory modes in an IVR environment. For learners with different prior levels of ability, signaling may have different advantages. This study provides further insight into the effect of multimedia design principles in IVR environments and delves into the effects of signaling and prior knowledge levels on learning effects in IVR experimental conditions.

(1) What are the effects of signaling and prior knowledge on the cognitive load of college students studying in an IVR environment?

(2) What are the effects of signaling and prior knowledge on the motivations of college students studying in an IVR environment?

(3) What are the effects of signaling and prior knowledge on the learning performance of college students studying in an IVR environment?

## 3. Materials and methods

### 3.1. Participants

A 2 × 2 factorial ANOVA design was used in this study, with the independent variables being signaling and prior knowledge, resulting in four experimental conditions: no signaling/low prior knowledge level, no signaling/high prior knowledge level, signaling/low prior knowledge level, and signaling/high prior knowledge level. The participants were recruited *via* the social media postings of a university. Forty volunteer undergraduate students (21 female and 19 male) from various majors at a large public university in the southwestern part of China participated in the study. All the participants completed and submitted informed consent forms. The age range of the participants was 21–30 years old (*M* = 23.975, *SD* = 1.60). The study used the HTC Vive Pro including a VR headset, base stations, controllers, mounting kit, etc. Therefore, participants were required to physically come to the laboratory to participate in the study. Due to the impact of the epidemic, 40 participants were recruited. Han et al. (2021) conducted a systematic review for IVR studies from 2004 to 2021, and the study reported that the sample sizes ranged from 8 to 174, with a median participant number of 58. Although the sample size is small, the research team added qualitative data, multiple data sources could help in answering the research questions (Twining et al., 2017).

### 3.2. Instruction materials

In this study, the laboratory course “Low-carbon Steel Tensile Experiment” of the architectural engineering technology major was used as the learning task in the IVR experiment. Unity 3D software was used in this study to build the IVR laboratory and conduct interactive design. The VR environment was composed of multiple simulation models. The VR laboratory environment in this study was simulated as an indoor environment. To simulate a sense of the environment, the lighting components of the software were used to simulate the lighting effect of a real environment. The computer used a camera to render and display the environment. This study simulated the IVR laboratory environment of architectural engineering technology through scene elements, which included game objects, directional light, and a unity camera. The design principles were as follows: (1) the simulated IVR environment should improve learners’ sense of immersion and presence in operation; (2) based on human-computer interactions, the operation steps in the IVR experiment should be consistent with those of the actual experiment; and (3) the human-computer interface should be both simple and practical to ensure smooth human-computer interactions.

**FIGURE 1:**
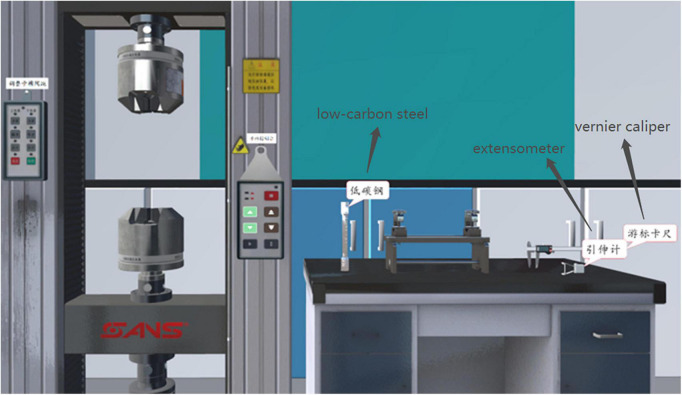
Text signals in the immersive virtual reality (IVR) experiment.

**FIGURE 2:**
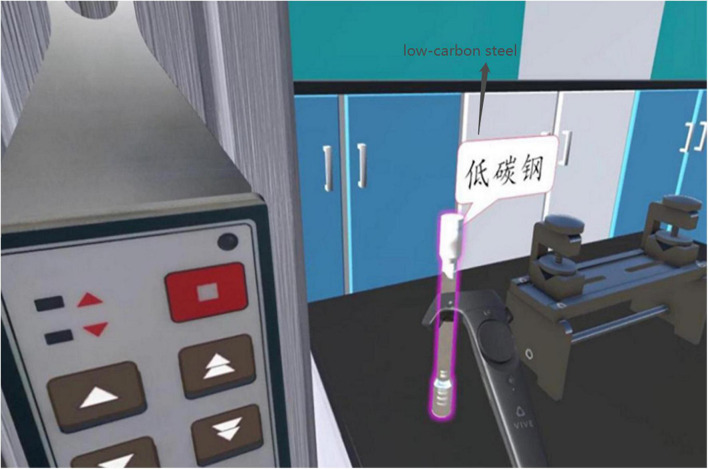
Highlight signals for the immersive virtual reality (IVR) experiment.

The main contents included observing four stages of the failure process of low-carbon steel, the shape of the truncated surface and the stress-displacement curve and understanding the relationship between the force and displacement to which the low-carbon steel is subjected during the tensile strength test. The experiment took 15–20 min to complete, and the five key steps were as follows: (1) correctly install the low-carbon steel on the electronic universal testing machine; (2) install the extensometer; (3) open the operating software, set parameters and fill in the preparatory information into the experimental report; (4) observe the low-carbon steel tensile test; and (5) fill in the results of the experimental report. The IVR experiment with signals is the same in learning content as that without signals, but the IVR experiment with signals has added text annotations (see Figure 1) and highlights the selected object (see Figure 2).

### 3.3. Instruments

#### 3.3.1. Prior knowledge test

The prior knowledge multiple-choice test included five questions related to the experimental contents of this study. The questions were used to test the participants’ basic knowledge of architectural mechanics related to the experiment, but no questions were posed that were similar to the experimental tasks to avoid influencing the subsequent experiments and tests. The test was positively scored, with higher scores indicating a higher level of prior knowledge of the learners. The questions on the prior knowledge test were compiled by the members of the research team based on relevant knowledge and were cross-reviewed by experts in architectural engineering technology for final confirmation.

#### 3.3.2. Cognitive load questionnaire

The NASA Task Load Index (NASA-TLX) scale was used in this study to measure the cognitive load of learners in completing the IVR experiment. The six dimensions measured by the scale were mental demand, physical demand, temporal demand, performance, effort, and frustration level. The Cronbach’s alpha of the scale was 0.85, indicating that the scale is a reliable assessment for measuring learners’ subjective mental workload.

#### 3.3.3. Intrinsic motivation scale

This intrinsic motivation scale, adapted from Ryan’s (1982) questionnaire and used in Lin and Li’s (2018) study, consisted of four subdimensions, including interest, competence, value, and pressure. The learners’ intrinsic motivation scale used a 7-point Likert scale, ranging from 1 (“Completely False”) to 7 (“Completely True”). Negative items were reverse scored, so higher scores reflect more positive motivation. The Cronbach’s alphas for interest, competence, value, and pressure were 0.90, 0.74, 0.78, and 0.76, respectively.

#### 3.3.4. Learning performance

After the four experimental groups finished the IVR experiment, they completed the learning performance post-test, which was set up as a question-type knowledge retention test. The six multiple-choice questions in this test were written by experts in architectural engineering technology and mainly included questions testing observation (e.g., “How many stages is the stress−strain diagram of low-carbon steel divided into during the tensile test?,” “What is the shape of the fracture of low-carbon steel?”), memory retention questions (e.g., “Please choose the correct sequence of low-carbon steel tensile experiments”), knowledge transfer questions (e.g., “What is the deformation of low-carbon steel like during its failure process before it is subjected to the maximum tensile stress? What does this deformation say about its tensile capacity?”), which were used to check whether the learners had mastered the knowledge related to the low-carbon steel tensile experiment.

### 3.4. Procedure

One week prior to the experiment, the participants received a knowledge pretest to assess their knowledge about the low-carbon steel experiment, and they were subsequently and accordingly grouped into high or low prior knowledge learners. A median score was used in this study to differentiate high from low levels of prior knowledge. Specifically, participants with test scores below or equal to the median score of the prior knowledge test (*Mdn* = 25) were grouped as having low prior knowledge (*n* = 20), and participants with test scores above the median score were grouped as having high prior knowledge (*n* = 20). The independent sample *t*-test revealed a significant difference in the mean scores of the pretest between the low prior knowledge group (*M* = 9.25, *SD* = 7.65) and the high prior knowledge group (*M* = 41.5, *SD* = 7.79), *t* = −13.20, *p* < 0.01. One week after taking the prior knowledge test, participants participated in the IVR experiment task to complete the learning and assessment parts of the study.

**FIGURE 3:**
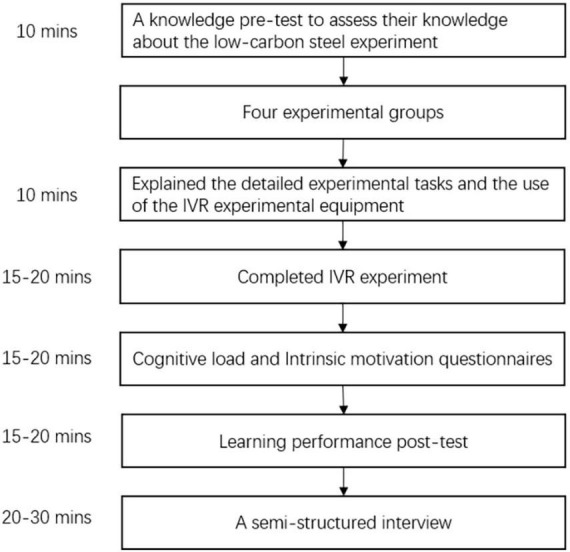
Experimental procedure.

The participants were semi-randomly assigned to no signaling (*n* = 20) and signaling (*n* = 20) conditions based on the results of the prior knowledge level pretest. The four experimental groups were as follows: no signaling/low prior knowledge level (*n* = 10), no signaling/high prior knowledge level (*n* = 10), signaling/low prior knowledge level (*n* = 10), and signaling/high prior knowledge level (*n* = 10). To carry out the experiment successfully, the participants first needed to be familiar with the knowledge related to the contents of the experiment. Reading materials for the low-carbon steel tensile experiment were provided by the researchers. Researchers explained the detailed experimental tasks and the use of the IVR experimental equipment so that the participants could be initially familiar with the requirements for operating virtual experimental environments and tasks. The IVR experiment was individually conducted for each participant. A research assistant helped participants to put on and adjust the HTC Vive Pro headset, and the participants began to learn and operate in an IVR laboratory environment. The learning content and tasks of the version with signals and those of the version without signals were the same. The version with signals included text annotations and object highlighting, which helped the learners select the experimental operation and access the key emphatic information of the selected object. Once preparations were complete, the experimental group and the control group wore HTC Vive Pro devices and started the IVR experiment. After the learners finished the low-carbon steel tensile strength experiment, they completed the learning performance test questionnaires, cognitive load scale, and intrinsic motivation scale and finally were led in a semi-structured interview. The quantitative data were analyzed by SPSS. The complete experiment time was approximately 100–120 min, and the experimental procedure is shown in Figure 3.

### 3.5. Interview

Three students were randomly selected from each of the four experimental groups, for a total of 12 students, and each student was interviewed for 20–30 min. Riches et al. (2019) proposed a theoretical framework with which to explore the factors affecting sense of presence in a VR environment, which included three dimensions: thoughts, emotions, and behaviors. Based on the theoretical framework, the interview questions comprised three categories. The first category involves the participants’ thoughts on signaling, e.g., are signals helpful to you? The second concerns the participants’ views on learning in an IVR environment, e.g., what parts of the IVR experiment scene appeal to you the most? The third is the comparison of the IVR laboratory with the traditional laboratory in terms of participants’ behaviors in the IVR, e.g., comparing the differences and making suggestions for improvement. The research team transcribed the interviews into text, and the three researchers coded the interviews and subsequently summarized them into themes. The three researchers compared the codes and themes and negotiated by presenting evidence (in the event of disagreements) to finally determine the following themes: operational feelings, recognition, and inadequacy.

## 4. Results

### 4.1. Effects of prior knowledge and signaling on cognitive load

To test whether signaling makes a difference in the cognitive load of learners with different prior knowledge levels, an ANOVA was conducted. The results showed that the main effect of signaling was not significant, *F*(3, 34) = 0.632, *p* = 0.432, η2 = 0.017, as shown in Table 1. The main effect of learners’ prior knowledge level was not significant, *F*(3, 34) = 0.044, *p* = 0.836, η2 = 0.001. The interaction effect between signaling and learners’ prior knowledge level was significant, *F*(3, 34) = 117.388, *p* = 0.000, η2 = 0.765, *p*-value less than 0.05. From the range of effect sizes, 0.14 or above indicated a large effect on the dependent variable. The effect size of learners’ prior knowledge level was 0.765, which is much larger than 0.14, thus indicating that the interaction effect of signaling and learners’ prior knowledge level in the IVR laboratory environment was the main factor causing significant changes in learners’ cognitive load. Specifically, for learners with a low prior knowledge level, signaling allows them to focus more on meaningful visual elements and reduces cognitive load in the IVR laboratory. On the other hand, learners with a high prior knowledge level had a lower cognitive load in the environment without signaling. Descriptive statistics showed that learners in the experimental condition without signaling (*M* = 4.283, *SD* = 1.347) had a higher cognitive load than those with signaling (*M* = 4.125, *SD* = 1.180), but the difference was not significant, as shown in Table 2. In terms of generated cognitive load, learners with a high prior knowledge level (*M* = 4.183, *SD* = 1.258) generated a lower cognitive load in the IVR laboratory than learners with a low prior knowledge level (*M* = 4.225, *SD* = 1.279), but the difference was not significant.

**TABLE 1:** Analysis of variance (ANOVA) results.

	Dependent variable	Type III sum of squares	df	Mean square	*F*	Sig	η^2^
Signaling	Cognitive load	0.251	1	0.251	0.632	0.432	0.017
	Intrinsic motivation	0.437	1	0.437	2.351	0.134	0.061
	Post-test	140.625	1	140.625	1.246	0.272	0.033
Prior knowledge	Cognitive load	0.017	1	0.017	0.044	0.836	0.001
	Intrinsic motivation	0.003	1	0.003	0.018	0.895	0.000
	Post-test	1,050.625	1	1,050.625	9.310	0.004	0.205
Signaling* prior knowledge	Cognitive load	46.584	1	46.584	117.388	0.000	0.765
	Intrinsic motivation	0.331	1	0.331	1.777	0.191	0.047
	Post-test	180.625	1	180.625	1.601	0.214	0.043

a. *R*^2^ = 0.766 (Adjusted *R*^2^ = 0.747). b. *R*^2^ = 0.103 (Adjusted *R*^2^ = 0.029). c. *R*^2^ = 0.252 (Adjusted *R*^2^ = 0.190).

*Interaction effect between signaling and learners’ prior knowledge level.

**TABLE 2:** Descriptive statistical analysis results.

	VR version	Prior knowledge	Mean	Standard deviation	*N*
**Intrinsic motivation**	Signal	Low	4.500	0.423	10
		High	4.300	0.479	10
		Total	4.400	0.452	20
	No signal	Low	4.109	0.475	10
		High	4.273	0.332	10
		Total	4.191	0.408	20
	Total	Low	4.305	0.481	20
		High	4.286	0.401	20
		Total	4.295	0.438	40
**Cognitive load**	Signal	Low	3.067	0.545	10
		High	5.1837	0.388	10
		Total	4.1257	1.180	20
	No signal	Low	5.383	0.4161	10
		High	3.183	0.983	10
		Total	4.283	1.347	20
	Total	Low	4.225	1.279	20
		High	4.183	1.258	20
		Total	4.204	1.252	40
**Post-test**	Signal	Low	37.00	6.749	10
		High	43.00	8.233	10
		Total	40.00	7.947	20
	No signal	Low	29.00	13.904	10
		High	43.50	12.030	10
		Total	36.25	14.679	20
	Total	Low	33.00	11.402	20
		High	43.25	10.036	20
		Total	38.13	11.804	40

### 4.2. Effects of prior knowledge and signaling on intrinsic motivation

To test the different influences of signaling and prior knowledge level on learners’ intrinsic motivation in the IVR laboratory, an ANOVA was conducted with the intrinsic motivation of the subjects as the dependent variable. The results showed that the main effect of learners’ prior knowledge level was not significant, *F*(3, 34) = 0.018, *p* = 0.895, η2 = 0.000, *p*-value greater than 0.05. The main effect of signaling on intrinsic motivation was not significant, *F*(3, 34) = 2.351, *p* = 0.134, η2 = 0.061, *p*-value greater than 0.05. The interaction effect between signaling and learners’ prior knowledge level was not significant, *F*(3, 34) = 1.777, *p* = 0.191, η2 = 0.047, *p-*value greater than 0.05. While descriptive statistical analysis revealed that learners with low prior knowledge levels were more motivated to learn (*M* = 4.305, *SD* = 0.481) than learners with high prior knowledge levels (*M* = 4.286, *SD* = 0.401), the difference was not significant. The results of the descriptive statistical analysis showed that while the IVR laboratory with signaling (*M* = 4.400, *SD* = 0.452) was more likely to stimulate learners’ intrinsic motivation than that without signaling (*M* = 4.191, *SD* = 0.408), the difference was not significant. The results of the data analysis indicated that there was no significant difference between the conditions of signaling and prior knowledge level in the IVR laboratory in terms of triggering learners’ intrinsic motivation, which remained at a high level.

### 4.3. Effects of prior knowledge and signaling on learning

To test whether signaling can affect learning performance and whether it differently affects learners who have different prior knowledge levels, an ANOVA was conducted with the participants’ learning performance as the dependent variable. The results show that the main effect of learners’ knowledge and experience level was significant, *F*(3, 34) = 9.310, *p* = 0.004, η2 = 0.205, *p*-value less than 0.05. The presence of signaling was not significant, *F*(3, 34) = 1.246, *p* = 0.272, η2 = 0.033, *p*-value greater than 0.05. The interaction effect of signaling and learners’ prior knowledge level was not significant, *F*(3, 34) = 1.601, *p* = 0.214, η2 = 0.043, *p*-value greater than 0.05. At the same time, based on effect size, learners’ prior knowledge levels in the IVR laboratory were the main factor causing changes in learners’ learning performance with a large effect size of 0.205, which was above 0.14. This result indicated that changes in learning performance were mainly influenced by learners’ prior knowledge level, and there was no significant difference between the presence of signaling and the interaction effect of signaling and the learners’ prior knowledge level on their learning performance. Based on the descriptive statistical analysis results, prior knowledge level had a more significant effect on the subjects’ learning performance in the IVR laboratory, and learners with a high prior knowledge level (*M* = 43.25, *SD* = 10.036) obtained better learning performance compared to the post-test performance of learners with a low prior knowledge level (*M* = 33.00, *SD* = 11.402). In the IVR laboratory with signaling (*M* = 40.00, *SD* = 7.947) learners achieved higher learning performance than those in that with no signaling (*M* = 36.25, *SD* = 14.679), but the difference was not significant.

### 4.4. Qualitative results

This study adopted the directed qualitative analysis method, which is guided by a more structured process than in a conventional approach (Hickey and Kipping, 1996). Directed qualitative analysis is a more appropriate approach when there are already existing theories about the phenomenon under investigation (Hsieh and Shannon, 2005). Based on Riches et al. (2019)’s theoretical framework, the study reported three themes: (1) learners’ operational feelings about the virtual experiment (thoughts), (2) learners’ attitudes of the virtual experiment (emotions), and (3) based on learners’ behaviors and experiences in VR, their opinions about the inadequacies of the virtual experiment.

Regarding learners’ operational feelings about the virtual experiment, the students in the IVR laboratory environment with signals reported that the “object highlighting” was very helpful for experimentation and that there were some differences between experimentation in the virtual environment and the real scene, mainly in the way the objects were touched. In this study, the IVR controller was activated to pick up and move the object and select the button. Some students said, for example:

*“The object highlighting is a quick way to determine whether the object has been touched*.”

They were able to operate more smoothly than learners in an IVR laboratory environment without signals, avoiding the impact of uncertainty on experimental procedures. Some learners also found the “text annotation” signal helpful, and this was generally true for learners with a low prior knowledge level. These less experienced learners thought the text annotations were intuitive and concise and found it easy to understand and proceed to the next step due to the clear cues throughout the experiment. However, a small number of students with a high prior knowledge level thought that the text annotations interfered with their own thinking and implementation of the experimental procedure, for example:

“*I was thinking about what to do next, but the text annotations interrupted my thinking*.”

Many students said, for example:

“*The content of the experiment is very interesting, and it is a novel experience to be able to conduct experiments virtually*.”.

This is because most of the learners were performing an IVR experiment for the first time and had a great interest in IVR. Therefore, prior knowledge level and the VR version had little effect on the intrinsic motivation of learners, and learners were very willing to perform similar experimental operations in immersive virtual scenes. Six students with high prior knowledge levels reported that the design of the virtual laboratory scenes was very realistic, for example:

“*The electronic universal testing machine, Vernier callipers and other experimental instruments are vividly rendered, and the setting of the laboratory makes people feel the authenticity of the scene, with a strong sense of situational immersion*.”

Thus, the sense of immersion is an important factor in stimulating learners’ interest and motivation. Object highlighting, text annotations and experimental content are the main factors that affect learners’ implementation of the procedures in the IVR laboratory. These signals further affect the extraneous cognitive load and intrinsic motivation of learners during the experiments, while different signals in the experiments influence learners’ cognitive processes and determine whether they are alert to the details of low-carbon steel stretching during the experiment (e.g., low-carbon steel failure process, fracture shape, etc.), thus impacting the post-test learning performance.

Regarding learners’ attitudes of the virtual experiment, most of the students in the interviews affirmed the effectiveness of the immersive virtual environment designed in this study in the following ways: (1) They thought the experiment was interesting. Some students said, for example:

“*I like operating the experimental equipment in the virtual scene*” and “*It made me want to understand how this experiment works*.”

(2) It contributes to inquiry-based learning. For example, a student stated the following:

“*I can control my own learning pace by doing experiments in this kind of virtual scene, and I can repeat steps that I am not familiar with*.”

(3) It is helpful for observation and memory. For example, a student mentioned the following:

“*In the immersive virtual laboratory, I am very relaxed while learning the content and can observe the experimental phenomena at close range to form a strong memory of knowledge such as the fracture section shape of low-carbon steel when it was stretched*.”

(4) They are willing to learn the experiment again. Most students believed that doing the experiment was of certain value to themselves and expressed their willingness to complete the experiment in an IVR laboratory. According to the overall analysis, the IVR laboratory had a relatively positive impact on the learners, which in turn stimulated their motivation and interest in learning.

Regarding inadequacies of the virtual experiment, by sorting out the questions raised by the interviewees about the IVR experiment, the following two reflections on inadequacies are proposed: (1) In terms of VR technology, IVR technology can provide high-quality display resolution, a wider field of view and an experience that stimulates multiple sensory modalities, but at the same time, this experience may bring physiological discomfort to some participants, such as those with vertigo. In this experiment, the IVR controller was used to achieve movement in the virtual scene, and the sensitivity of the IVR controller led to a certain discomfort caused by the fast roaming speed in the scene, which had the potential to produce vertigo. Vertigo would affect the whole experience of the IVR laboratory and might increase the extraneous cognitive load of the learners. The HTC headset would cause a certain discomfort for some learners who wear glasses, and at the same time, its weight would make the students uncomfortable. (2) In this particular experiment, learners who were unfamiliar with the low-carbon steel stretching experiment itself (learners with a low prior knowledge level) were more focused on the operation of the VR itself in the virtual environment and might have overlooked some details of the experiment that should have been observed. Some learners reported that there was a gap between their spatial cognition ability in the virtual scene and their ability in real life, and they could not distinguish left from right, which affected their own operation and judgment.

## 5. Discussion

This study explores the effects of signaling and different prior knowledge levels (high vs. low) on learners’ cognitive load, intrinsic motivation, and learning performance in the IVR laboratory. Several findings have been presented to support that the factor of signaling has a significant effect on reducing learners’ cognitive load (e.g., Arslan-Ari, 2018; Albus et al., 2021). This study found that the interaction effect of signaling and prior knowledge level on learners’ cognitive load in the IVR laboratory was significant and that an expertise/experience reversal effect on cognitive load emerged, i.e., signaling had a negative effect on the cognitive load of learners with high prior knowledge levels but had a positive effect on that of learners with low prior knowledge levels. The empirical findings of this study demonstrate that signaling in the IVR laboratory can effectively help learners with low prior knowledge operate experiments and observe experimental phenomena, providing greater support for reducing learners’ cognitive load. The findings provide suggestions and guidance for the design of IVR experiments and their application in teaching practice.

### 5.1. Effect of signaling

This study analyses the effects of signaling on students in an IVR laboratory and finds no significant differences in cognitive load, intrinsic motivation, or learning performance between the two groups of participants with and without signaling. From the results of multivariate ANOVA, in the analysis of the effects of only signaling, learners’ learning performance is higher in the VR version with signals (*M* = 40.00, *SD* = 7.947) than in the VR version without signals (*M* = 36.25, *SD* = 14.679), learners’ intrinsic motivation is higher in the VR version with signals (*M* = 4.400, *SD* = 0.452) than without signals (*M* = 4.191, *SD* = 0.408), and learners’ cognitive load is lower in the VR version with signals (*M* = 4.126, *SD* = 1.180) than without signals (*M* = 4.283, *SD* = 1.347), but there is no significant difference in the effect of signaling in the IVR laboratory on learning performance, intrinsic motivation and cognitive load.

This result is inconsistent with cognitive load theory and the cognitive–emotional theory of multimedia learning, both of which suggest that designing signals in multimedia learning materials that are highly relevant to the teaching content can help learners reduce visual retrieval and lower cognitive load (Fiorella and Mayer, 2014). However, there are still some researchers whose findings are consistent with the present study. Nelson et al. (2014) hypothesized that the use of visual signals could reduce the overall cognitive load of students when completing virtual world-based tasks, yet the findings showed only small significant differences in cognitive load between the groups. The reason given in their analysis was that the virtual scenes themselves were designed to contain fewer interactive objects and limited virtual space for exploration, which has been found to reduce learners’ cognitive load. It has also been suggested that signaling only improves learning performance with complex learning materials (Albus et al., 2021). Similarly, Jeung et al. (1997) suggested that adding visual signals to learning materials that required high levels of visual searching facilitated learning, while adding visual signals to conditions with low levels of visual searching had little effect on learning. The IVR experiment scene designed in this study is as simple and direct as possible, with no redundant or irrelevant scene elements. Thus, the visual impact on learners does not reach the level of so-called “complexity” that would increase cognitive load. However, it is also clear that the design of IVR laboratories should not be overloaded with visual overlays, as this would result in a visual input burden and cognitive processing overload for learners. This suggests that the application of signaling needs to be designed in conjunction with the actual virtual scene to provide the most concise and effective guidance for learners.

In terms of the types and content of signaling, this study uses signals in the form of textual annotations and color cues, which allow learners to receive signals (Baceviciute et al., 2020) in a visually demanding virtual environment through short and concise signals. Knowing which information is relevant now and which needs to be looked at more closely can reduce unnecessary searches and draw attention to relevant aspects. However, as some of the text involves technical terms (e.g., manual control box, fixture, etc.), it can be difficult for learners with a low prior knowledge level to understand, and they need to invest mental effort in the operation of the experiment, thus potentially increasing the cognitive load and impacting learning effects. Furthermore, the findings of Richter et al. (2016) suggest that compared with multimedia integrated signals, learning materials with a more prominent visual appearance, such as color coding, may be more accessible to learners than discursive signals as well as descriptive references. In this study, signals are presented in the form of dialog boxes in the scene. The study could have better embedded the signals into the environment, and learners’ sense of learning experience may have been affected (Albus et al., 2021). Thus, in signal design, the learning situation can be combined with the laboratory design, dynamic signals can be combined with static signals, different depths and elaborations of the integration of text and images can be made, and other multimedia design principles, such as the redundancy principle and the spatial proximity principle, can be combined to provide good signal effects for learners (Lai et al., 2019).

### 5.2. Effect of prior knowledge level

It is found that learners’ prior knowledge level plays an important role in influencing learning performance, intrinsic motivation and cognitive load in IVR laboratories. From the results of multivariate ANOVA, in the analysis of only the effect of learners’ prior knowledge level, the learning performance of learners with a high prior knowledge level (*M* = 43.25, *SD* = 10.036) is significantly higher than that of learners with a low prior knowledge level (*M* = 33.00, *SD* = 11.402), indicating that prior knowledge has a significant impact on learning performance in an IVR laboratory. Intrinsic motivation was slightly lower in learners with a high prior knowledge level (*M* = 4.286, *SD* = 0.401) than in learners with a low prior knowledge level (*M* = 4.305, *SD* = 0.481). Cognitive load was slightly lower in learners with a high prior knowledge level (*M* = 4.183, *SD* = 1.258) than in learners with a low prior knowledge level (*M* = 4.225, *SD* = 1.279), indicating that learners with high prior knowledge levels had higher learning performance and lower cognitive load overall when learning in the immersive virtual laboratory, which is a finding consistent with most studies (Kalyuga et al., 2004; Kriz and Hegarty, 2007). Another study showed that learners’ attitude-learning-confidence is strongly related (Taçgın, 2020) and that learners with high prior knowledge levels have more positive attitudes and interest in learning in virtual learning environments. In summary, it is recommended that novice learners familiarize themselves with the IVR technology before using the system as a learning tool and improve their learning effects by increasing their confidence or attitude with repeated practice. These findings are almost unanimous in studies that address only the effect of prior knowledge level on learning effects, but with the addition of some intervening factors for moderation, there may be an interaction effect, and the results may vary.

### 5.3. Interaction effect of prior knowledge level and signaling

Signaling is able to help learners with low prior knowledge levels reduce their cognitive load generated by completing the immersive virtual experiment task, enhance their intrinsic motivation, and achieve better performance on the final test, while learners with high prior knowledge levels had better learning performance without these additional signals (Kalyuga, 2007; Richter et al., 2016). The results of the data analysis support the hypothesis that the effectiveness of signaling in the IVR laboratory differs for learners with different prior knowledge levels, indicating that the expertise reversal effect of signaling on learners is as valid in an IVR laboratory as in the traditional multimedia learning environment (Johnson et al., 2013, 2014; Khacharem, 2017; Arslan-Ari, 2018). Therefore, in conducting IVR laboratories, it is quite effective to direct the attention of learners with low prior knowledge levels to help them select relevant information. From this study, the design of synchronous textual signals on the microcomputer-controlled electronic universal testing machine used for completing experimental tasks can direct learners’ attention to the aspects of the scene related to conducting the experiment. Thus, these synchronous signals reduce external load and help learners use their mental resources to build coherent mental representations of the target learning material and integrate these representations into long-term memory (Arslan-Ari, 2018). In addition to text signals, color changes are used as signals in this study, which play an important role in IVR experiments. Based on learner feedback, the largest difference between immersive and realistic laboratories is the selection of relevant objects. Most existing virtual reality technology uses the IVR controller for interaction, and learners have no real sense of touch when they interact with the corresponding scene elements (Streppel et al., 2018). There can be confusion about whether they have picked up the object, which affects the thinking and cognitive load and can also reduce the learning experience. Using color changes to signal that an object has been touched helps learners carry out the operation steps smoothly and coherently. Multimedia learning cognitive–affective theory, cognitive load theory and other theories on media learning consider the learner’s prior knowledge base to be the most important moderator. Previous studies have proven that signal transmission can enhance students’ knowledge retention ability (Boucheix et al., 2013) and knowledge transfer ability in problem-based learning (Ouwehand et al., 2014). Although the interaction effect of the two is not significant for learners’ intrinsic motivation and learning performance, the results of the data analysis suggest that the joint effect of signaling and prior knowledge level on learners is an area worthy of more research and an existing research trend (Arslan-Ari, 2018). Learners with low prior knowledge levels are slightly more intrinsically motivated than learners with high prior knowledge levels in the condition with signaling, while in the condition without signaling, learners with high prior knowledge had higher intrinsic motivation.

The results of this study also demonstrate the need for personalized learning and the need for developers to adopt a different mindset in the design and development of immersive virtual experiments to cater to differences between learners with different prior knowledge levels and other characteristics (Kalyuga, 2007). Based on the above findings and the characteristics of immersive virtual laboratories, developers need to reintegrate the association between the prior knowledge level and signaling (Kalyuga, 2008), design signal types that are highly relevant to the experimental content and virtual scenes, tailor the available information to the prior knowledge levels of target students, and ensure that the new material is consistent with their original mental representations. VR designers could offer VR participants the option to toggle between signals and no-signals in the same way that a TV viewer can toggle between closed captioning and no closed captioning. If a student is getting distracted/annoyed by the dialog boxes in this low carbon steel tensile strength experiment, for example, they could just opt to disable the dialog boxes.

## 6. Conclusion and implications

The core purpose of this study is to explore the effect of signaling and prior knowledge on learning effects in an IVR laboratory. This study suggests that the design of IVR experiments should be combined with the characteristics of different learners. For learners with high prior knowledge levels, only the necessary signals should be designed and offered as resources, as additional signals will result in higher cognitive load with a negative effect on the learning experience and potentially the final learning performance. When learners’ prior knowledge is low, appropriate signals can help to reduce cognitive load and improve learning performance. Future research may recruit more participants to obtain a better effect size. The study applied NASA-TLX to measure participants’ cognitive load. Despite being widely used as a measure of cognitive load, the NASA-TLX has several limitations. More empirical research on measuring cognitive load is needed. Future empirical studies should also consider using multimodal channels to measure learners’ cognitive load, such as EEG, ECG, EDA, DDT, and eye tracking, which could more accurately gather data and deeply explore the mechanisms related to cognitive load. The IVR experimental scene in this study is designed with only two types of signals, text annotation and color, and future research can further analyze the effects of signal type, complexity, level of detail, and information content on learners.

## Data availability statement

The raw data supporting the conclusions of this article will be made available by the authors, without undue reservation.

## Ethics statement

The studies involving human participants were reviewed and approved by the Dr. Shengquan Luo, Faculty of Education, Southwest University. The patients/participants provided their written informed consent to participate in this study. Written informed consent was obtained from the individual(s) for the publication of any potentially identifiable images or data included in this article.

## Author contributions

JH: conceptualization, investigation, methodology, data collection, writing – review and editing, and validation. GL: conceptualization, writing – review and editing, and supervision. QZ: data collection and formal analysis. All authors have read and agreed to the published version of the manuscript.
